# Ethylene and auxin interaction in the control of adventitious rooting in *Arabidopsis thaliana*

**DOI:** 10.1093/jxb/erw415

**Published:** 2016-11-09

**Authors:** A. Veloccia, L. Fattorini, F. Della Rovere, A. Sofo, S. D’Angeli, C. Betti, G. Falasca, M.M. Altamura

**Affiliations:** ^1^Dipartimento di Biologia Ambientale, Sapienza Università di Roma, Roma, Italy; ^2^School of Agricultural, Forestry, Food and Environmental Sciences (SAFE), Università degli Studi della Basilicata, Potenza, Italy; ^3^Department of Plant Biotechnology and Bioinformatics, Ghent University, 9052 Gent, Belgium; ^4^Department of Plant Systems Biology, VIB, 9052 Gent, Belgium

**Keywords:** Adventitious roots, *Arabidopsis thaliana*, auxin, IAA-biosynthesis and transport, ethylene signaling, IBA-to-IAA conversion, IBA transport.

## Abstract

Adventitious roots (ARs) are post-embryonic roots essential for plant survival and propagation. Indole-3-acetic acid (IAA) is the auxin that controls AR formation; however, its precursor indole-3-butyric acid (IBA) is known to enhance it. Ethylene affects many auxin-dependent processes by affecting IAA synthesis, transport and/or signaling, but its role in AR formation has not been elucidated. This research investigated the role of ethylene in AR formation in dark-grown *Arabidopsis thaliana* seedlings, and its interaction with IAA/IBA. A number of mutants/transgenic lines were exposed to various treatments, and mRNA *in situ* hybridizations were carried out and hormones were quantified In the wild-type, the ethylene precursor 1-aminocyclopropane-1-carboxylic acid (ACC) at 0.1 μM enhanced AR formation when combined with IBA (10 μM), but reduced it when applied alone; this effect did not occur in the *ein3eil1* ethylene-insensitive mutant. ACC inhibited the expression of the IAA-biosynthetic genes *WEI2*, *WEI7*, and *YUC6*, but enhanced IBA-to-IAA conversion, as shown by the response of the *ech2ibr10* mutant and an increase in the endogenous levels of IAA. The ethylene effect was independent of auxin-signaling by TIR1-AFB2 and IBA-efflux by ABCG carriers, but it was dependent on IAA-influx by AUX1/LAX3.

Taken together, the results demonstrate that a crosstalk involving ethylene signaling, IAA-influx, and IBA-to-IAA conversion exists between ethylene and IAA in the control of AR formation.

## Highlight

Ethylene affects adventitious rooting by reducing indole-3-acetic acid (IAA) biosynthesis, but enhancing conversion into IAA of its precursor indole-3-butyric acid (IBA). This conversion, together with active IAA-cellular-influx, is essential for adventitious root formation.

## Introduction

Ethylene is a gaseous hormone involved in many aspects of plant development. It is biologically active in trace amounts, diffuses from cell to cell across membranes, and is produced by all plant cells (Bleecker and Kende, 2000; Lin *et al.*, 2009). Methionine gives rise to ethylene via its conversion to S-adenosyl-L-methionine, which, in turn, is converted to 1-aminocyclopropane-1-carboxylic acid (ACC) by ACC synthase (ACS), followed by ACC degradation by ACC-oxidase (ACO).

Being the direct ethylene precursor, ACC is exogenously applied as an experimental treatment for investigating ethylene responses (Ivanchenko *et al.*, 2008, and references therein). It is known that when treated with ethylene, or ACC, the resulting etiolated seedlings of Arabidopsis show the triple-response phenotype, i.e. inhibition of the primary root (PR) and hypocotyl elongation, radial swelling of the hypocotyl and PR, and exaggeration in the curvature of the apical hook (Ecker, 1995). The absence of this phenotype in specific mutants has allowed the identification of key genes in ethylene response/signaling (Wang *et al.*, 2013, and references therein).

Many processes under ethylene control are also affected by indole-3-acetic acid (IAA). The two hormones act synergistically in inhibiting PR elongation, with ethylene involved in regulating the transcription of the PIN1, PIN2, and PIN4 IAA-efflux carriers, and the AUX1 influx-carrier (Růžička *et al.*, 2007; Swarup *et al.*, 2007). Moreover, the two hormones affect each other’s synthesis, because high auxin levels lead to increased ethylene synthesis, through an increased transcription of ethylene synthesis genes, such as *ACS* genes (Muday *et al.*, 2012). In turn, in the Arabidopsis primary root, ethylene induces the *WEAK ETHYLENE-INSENSITIVE2/ANTHRANILATE SYNTHASE alpha1* (*WEI2/ASA1*) and *WEI7/ANTHRANILATE SYNTHASE beta1* (*ASB1*) genes that encode, respectively, the α- and β-subunits of anthranilate synthase, which is a rate-limiting enzyme of an early step of tryptophan (Trp)-dependent IAA biosynthesis (Stepanova *et al.*, 2005). It has also been demonstrated that ethylene affects auxin signaling mediated by TIR1 (TRANSPORT INHIBITOR RESPONSE1) (Muday *et al.*, 2012, and references therein).

Adventitious roots (ARs) are post-embryonic roots that arise from the plant aerial organs and the non-pericycle tissues of the PR (Fahn, 1990). ARs are necessary for survival in numerous plants, they are essential for vegetative propagation *in planta* and *in vitro*, and they are a determinant for breeding programs. In Arabidopsis, ARs originate from the pericycle of the hypocotyl, with formation favoured by seedling growth under continuous darkness (Takahashi *et al.*, 2003). Natural/synthetic auxins are widely known to positively affect AR formation (Falasca and Altamura, 2003; Pacurar *et al.*, 2014). In Arabidopsis, IAA induces AR initiation through the activation of a coordinated efflux/influx involving PIN-FORMED1 (PIN1) and AUXIN1 (AUX1) that acts to cause the IAA gradient essential for induction. On the other hand, the influx carrier LIKE-AUX1-3 (LAX3) is essential for AR emergence (Della Rovere *et al.*, 2016). Moreover, the auxin flux caused by *PIN1*, *AUX1*, and *LAX3*, which is coordinated with local auxin biosynthesis by YUCCA6 (YUC6) that converts the indole-3-pyruvic acid produced from tryptophan into IAA (Brumos *et al.*, 2014), is necessary to produce the auxin maximum required for initiation/maintenance of the quiescent centre and niche cells at the tip of the AR (Della Rovere *et al.*, 2013).

Indole-3-butyric acid (IBA) is the AR-inducing auxin that is most used for *in vitro* cultured explants, because its root-inducing capacity is higher than that of IAA, which is due in part to its higher resistance to light-induced degradation compared with IAA (Bellini *et al.*, 2014). *In planta*, the auxin pool consists of free IAA, IAA conjugates, and IBA (Korasick *et al.*, 2013). IBA is the naturally occurring auxin precursor in many plants, including Arabidopsis (Simon and Petrášek, 2011). In Arabidopsis, there is evidence that IAA and IBA use distinct cellular transport systems, that IBA is inactive during its cell-to-cell transit, and that IBA activity completely depends on its conversion to IAA in the target cells (Strader and Bartel, 2011; Sauer *et al.*, 2013).

The role of ethylene in AR formation has been examined in a variety of plants, but the results have been contradictory, with both positive and negative effects reported even in the same species (Negi *et al.*, 2010, and references therein). This research was aimed at investigating the role of ethylene in AR formation in dark-grown *Arabidopsis thaliana* seedlings, and at testing the hypothesis that AR formation is controlled by the interaction of ethylene with the two main natural auxins, i.e. IAA and IBA, through interferences with reciprocal synthesis, transport, and/or signaling.

To achieve this aim, experiments were designed following an integrated approach. First, the adventitious rooting response to ACC and/or IBA was evaluated in mutants for genes involved in ethylene signaling (*ein3eil1*; Alonso *et al.*, 2003; Zhu *et al.*, 2011), and in IAA signaling (*tir1afb2*; Parry *et al.*, 2009), biosynthesis (*wei2-1wei7-1*; Stepanova *et al.*, 2005), and transport (*lax3aux1*; Della Rovere *et al*., 2013, 2015). Next analyses of IBA-to-IAA conversion (*ech2-ibr10*; Strader *et al.*, 2011) and IBA-cellular-efflux (*abcg36abcg37*; Strader and Bartel, 2011) were combined with analyses of the localization of IAA-induced gene expression (*DR5::GUS*) and transport (*PIN1::GUS*, *AUX1::GUS*, *LAX3::GUS*). Finally, the IAA biosynthetic gene expression (*YUC6*, *ASA1*, *ASB1*) was determined, together with quantifications of IAA and IBA.

The results provide evidence about the mechanisms of regulation of AR formation by ethylene, and the crosstalk between ethylene, IAA, and its precursor IBA in the control of the process.

## Materials and methods

### Plant material

*Arabidopsis thaliana* Col and Col-0 ecotypes formed the background/wild-type (WT) for the transgenic lines and mutants used in this research.

The following genotypes were used in this study: *DR5::GUS*, *PIN1::GUS*, *AUX1::GUS*, *LAX3::GUS*, *ASA1::GUS*, *ASB1::GUS*, *lax3aux1-21*, *abcg36-4abcg37-2*, *ein3eil1*, *ech2-1ibr10-1*, *wei2-1wei7-1*, and *tir1-1afb2-3*, together with Col and Col-0. Stocks of 150 seeds per genotype were surface-sterilized and sown on Petri plates (12 seeds per plate) containing MS (Murashige and Skoog, 1962) salts, 0.55 mM myo-inositol, 0.1 μM thiamine-HCl, 1% (w/v) sucrose, and 0.8% (w/v) agar (Della Rovere *et al.*, 2013), either without hormones (hormone-free, HF), or with ACC alone (0.01, 0.04, 0.1, or 1 μM for the WT, and 0.1 μM for the other genotypes), with IBA alone (10 μM according to Ludwig-Müller *et al.* 2005), or with IBA plus ACC (10 μM and 0.1 μM, respectively). After stratification for 3 d at 4 °C under continuous darkness and exposure to white light (intensity 100 μEm^−2^ s^−1^) for 6 h to induce seed germination, the plates were placed in a vertical position under continuous darkness at 22 ± 2 °C, according to Takahashi *et al.* (2003). At day 7 after stratification (DAS), the percentage of seed germination was calculated in the WT under all the culture conditions. At 22 DAS, the final response was evaluated in all genotypes. Chemicals were provided by Sigma-Aldrich.

### Histological and histochemical analysis

At 22 DAS, 30 seedlings per genotype/treatment were fixed in 70% ethanol solution.

Hypocotyl length was measured under a Leica MZ8 stereomicroscope using the software Axio Vision Release 4.7.2 and a Zeiss AxioCam camera. AR primordia (ARPs) and ARs were counted under a Leica DMRB microscope in bright field mode, and images were captured with a Leica DC 500 camera (Leica IM1000 Image Manager Software). AR number was expressed as mean density per cm of hypocotyl.

Seedlings of all the GUS lines were processed for β-glucuronidase (GUS) staining according to Willemsen *et al.* (1998), and incubated at 37 °C in the dark either for 30 min (*DR5::GUS* and *LAX3::GUS*), 45 min (*AUX1::GUS*, *ASA1::GUS*, and *ASB1::GUS*), or 2.5 h (*PIN1::GUS*). Finally, the GUS buffer was replaced by 70% ethanol until observation. The observations were carried out with a Leica DMRB microscope in bright field mode, and images were captured with a Leica DC 500 camera and the software Leica IM1000 Image Manager. The pattern of the GUS signal was confirmed by more than 90% of the observations per transgenic line.

### *Whole-mount* in situ *hybridization*

At 22 DAS, 30 Col-0 seedlings from each treatment (HF, 0.1 μM ACC, 10 μM IBA, 10 μM IBA + 0.1 μM ACC) were collected, fixed, stored, and treated with digoxigenin-labelled *YUC6* antisense and sense RNA probes, and then mRNA was detected according to Della Rovere *et al.* (2013).

The seedlings were observed under a Leica DMRB microscope in bright field mode. Thirty ARs per treatment were examined, and the pattern of the hybridization signal in the ARs was confirmed by more than 90% of the observations. The absence of a hybridization signal in the samples treated with the sense probe was also verified.

### Hormone quantification

At 22 DAS, about 100 seedlings of the Col-0, *ein3eil1*, and *tir1afb2* genotypes grown on HF, 0.1 μM ACC, 10 μM IBA, or 10 μM IBA + 0.1 μM ACC were collected and the hypocotyls with ARs were immediately stored at −80 °C. One hundred Col-0 seedlings were also grown on HF +/–ACC up to 14 DAS. The extraction of IAA and IBA was performed according to Pan *et al.* (2010) with minor modifications. Briefly, aliquots of 100 mg of hypocotyls with ARs were homogenized in a mortar on ice with 1 ml of 2-propanol/H_2_O/HCl 37% (2:1:0.002, v/v/v). To each sample, 1 ml of dichloromethane was added, and the samples were subsequently centrifuged at 13 000 *g* for 5 min at 4 °C. The lower phase was removed (750 μl), concentrated using an evaporator with nitrogen flow, and then re-dissolved in 15 μl methanol. Quantitative determinations of IAA and IBA were carried out by high-performance liquid chromatography coupled with mass spectrometry, according to Sofo *et al.* (2011) (Supplementary Fig. S1 at *JXB* online). Pure standards of the two hormones were used for quantification (Duchefa Biochemie B.V., Haarlem, The Netherlands). The internal standards used were [^2^H_5_] IAA and [^2^H_9_] IBA (OlChemIm Ltd, Olomouc, Czech Republic; crystalline form, purity >97% for HPLC). The amounts of IAA and IBA were determined by calculating the correction factor of each authentic hormone in comparison with its corresponding internal standard. Correction factors were calculated as the ratio of the signal intensity of the internal standard to the corresponding hormone.

### Statistical analysis

Data are expressed as means (±SE). One-way or two-way ANOVAs (*P*≤0.05) were used to compare the effects of treatments, or treatments and genotypes, respectively, and, if ANOVA showed significant effects, Tukey’s post-test was applied (GraphPad Prism 6.0). The significance of differences between percentages was evaluated using a *χ* ^2^ test. All the experiments were repeated three times in two consecutive years, and very similar results were obtained (data from the second year are shown in the text, and that from the first year are shown in Supplementary Figs S2 and S3).

## Results

A preliminarily evaluation of seed germination was made in the WT at 7 DAS in the presence of an ACC range from 0 to 1 μM, and in the presence of 0.1 μM ACC and/or IBA. No significant changes in germination were caused by ACC or IBA, thus excluding any effect on germination by the treatments (Supplementary Fig. S2A, C). The formation of ARs from the hypocotyls of seedlings of *Arabidopsis thaliana* Col-0 and Col was investigated at 22 DAS. The seedling growth period was prolonged by about 1 week in comparison with our previous studies under the same environmental conditions and WT genotype (Della Rovere *et al.*, 2013, 2015) in order to obtain a higher number of fully developed ARs. No significant differences between the WT genotypes were observed (Supplementary Fig. S4B)

### A specific ACC concentration inhibits AR formation in the absence of any exogenous hormonal input, but stimulates it in the presence of exogenous IBA

**Fig. 1.**
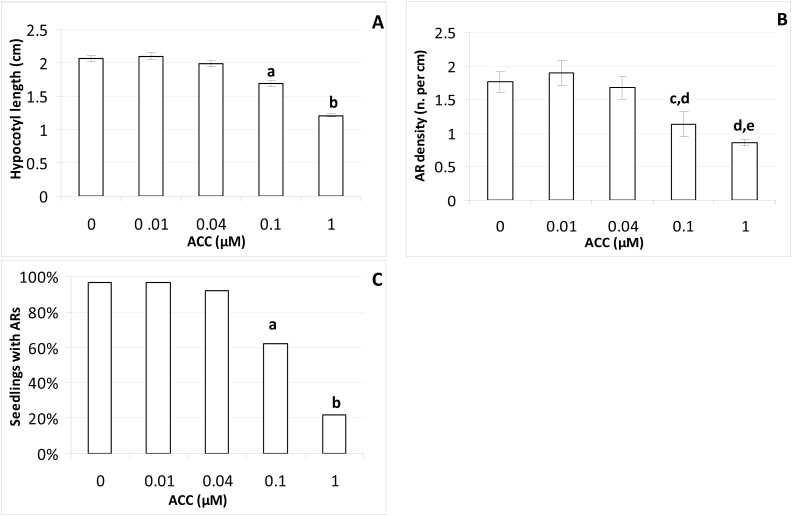
AR formation from hypocotyls of *Arabidopsis thaliana* seedlings, Col-0 ecotype, at the end of *in vitro* culture growth (22 DAS) under continuous darkness either in the absence of exogenous hormones (0, HF) or with different concentrations of ACC. (A), Mean hypocotyl length (±SE). (B) AR density, i.e. AR number per cm of hypocotyl, expressed as the mean value (±SE). (C) Percentage of seedlings with ARs. *n*=30. a, b, *P*<0.01 differences with respect to the other treatments; c, *P*<0.05 difference with respect to HF; d, *P*<0.01 difference with respect to 0.01 µM ACC; e, *P*<0.01 difference with respect to HF and 0.04 µM ACC. Columns with the same letter or no letter are not significantly different. Similar results were obtained with the Col ecotype.

Hypocotyl growth and AR formation were evaluated in the WT seedlings in the presence of a range of ACC concentrations from 0 to 1 μM. At 22 DAS the hypocotyl length was not significantly changed by any ACC concentrations lower than 0.1μM in comparison with the HF treatment, whereas at 0.1 μM there was a small, but significant, reduction (Fig. 1A). A drastic reduction (about 50%) was observed at 1 μM (Fig. 1A) but, differently from 0.1 μM, it occurred as part of the triple response (data not shown). ACC concentrations lower than 0.1 μM did not cause any effects on AR density and percentage of seedlings with ARs, whereas at 0.1 μM there was a significant reduction of both these parameters (Fig. 1B, C). The reduction in the percentage of seedlings with ARs was particularly enhanced at the highest ACC dose (1 μM) (Fig. 1C), but this seemed to be an indirect effect of the ACC-produced ethylene on the AR process linked to the drastic reduction in hypocotyl length (Fig. 1A). In accordance with this interpretation, the mean number of ARs produced per seedling reflected the results regarding AR density. In fact, 3.5 (±0.3), 3.9 (±0.4), and 3.3 (±0.3) ARs were produced under the HF, 0.01 μM, and 0.04 μM ACC treatments, respectively, with no significant difference among the treatments, whereas the mean AR number was reduced to 2.0 (±0.3) under 0.1 μM ACC (*P*<0.05 difference in comparison with 0–0.04 μM ACC) and it was further, and strongly, reduced under 1 μM (1.0 ± 0.1).

**Fig. 2.**
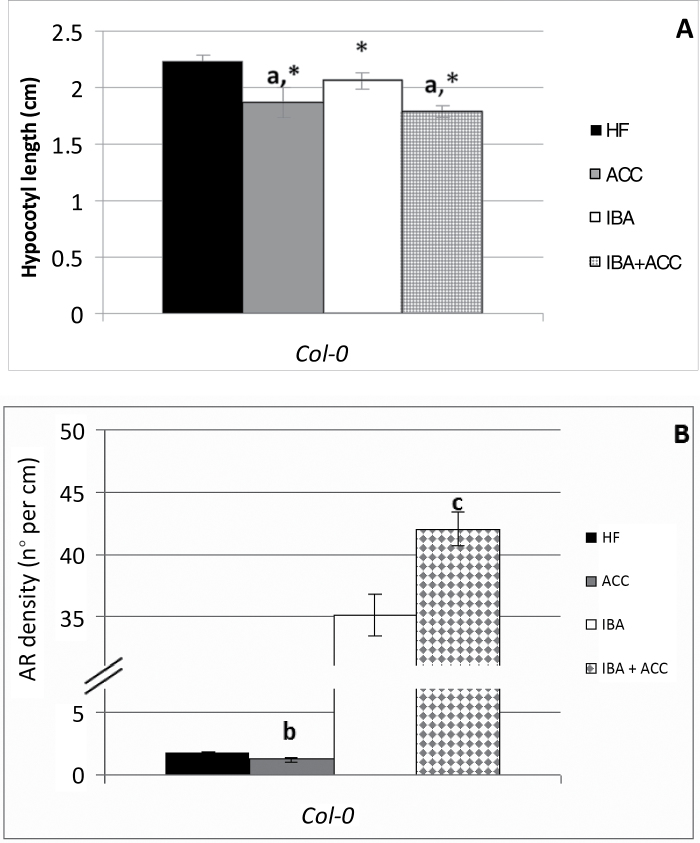
AR formation from hypocotyls of *Arabidopsis thaliana* seedlings of the Col-0 ecotype at the end of *in vitro* growth (22 DAS) under continuous darkness, either without hormones (HF) or with ACC (0.1 µM), and/or IBA (10 µM). (A) Mean hypocotyl length (±SE). (B) AR density, i.e. AR number per cm of hypocotyl, expressed as the mean value (±SE). *n*=30. a, *P*<0.01 difference with respect to HF; b, *P*<0.05 difference with respect to HF; c, *P*<0.01 difference with respect to IBA alone. Columns with the same letter or with an asterisk are not significantly different.

Based on these results, the WT seedlings were treated either with or without 0.1 μM ACC, combined either with or without 10 μM IBA. At 7 DAS, seed germination was not affected by these treatments (Supplementary Fig. S2C). At 22 DAS, ACC, IBA, and IBA+ACC caused a significant and comparable reduction of the hypocotyl length in comparison with the HF treatment (Fig. 2A and Supplementary Fig. S4A). AR density was significantly decreased by ACC alone, by 1.5-fold, and increased many-fold by IBA alone (*P*<0.01; 19-fold in Fig. 2B and 17-fold in Supplementary Fig. S4B). Surprisingly, IBA+ACC caused a significant further enhancement (1.2-fold) of AR density in comparison with IBA alone (Fig. 2B and Supplementary Fig. S4B).

### *The response of the* ein3eil1 *mutant suggests the involvement of ethylene signaling in AR formation*

**Fig. 3.**
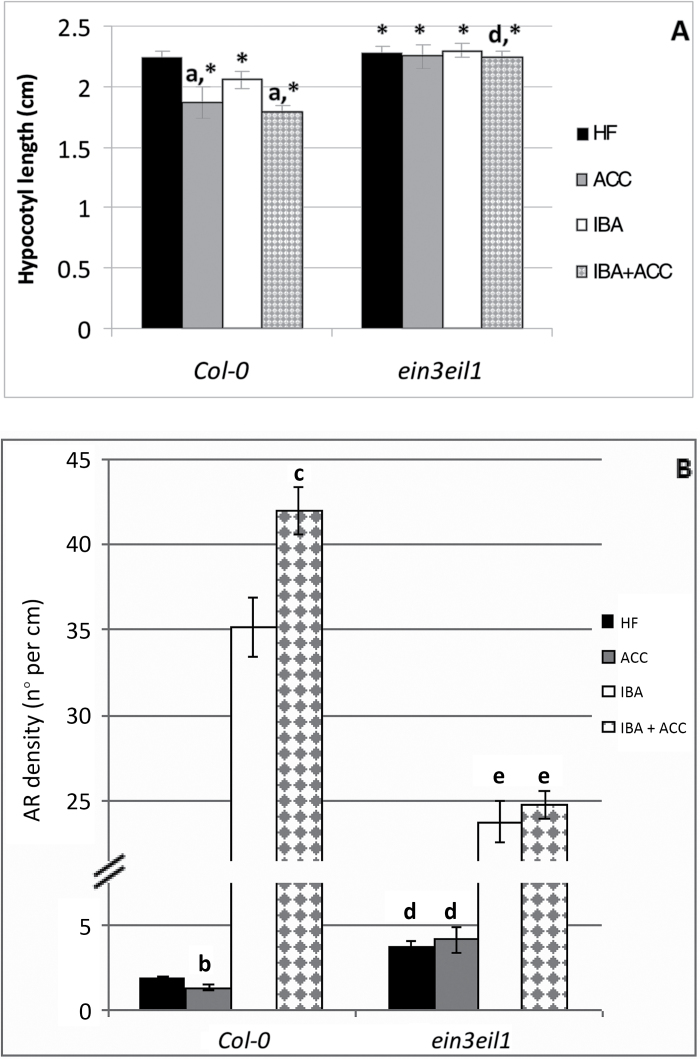
AR formation from hypocotyls of *Arabidopsis thaliana* seedlings of the Col-0 ecotype (WT) and of the *ein3eil1* double-mutant at the end of *in vitro* growth (22 DAS) under continuous darkness either without hormones (HF) or with ACC (0.1 µM) and/or IBA (10 µM). (A) Mean hypocotyl length (±SE). (B) AR density, i.e. AR number per cm of hypocotyl, expressed as the mean value (±SE). *n*=30. a, *P*<0.01 difference with respect to HF within the same genotype; b, *P*<0.05 difference with respect to HF within the same genotype; c, *P*<0.01 difference with respect to IBA within the same genotype; d, e; *P*<0.01 difference with respect to the WT within the same treatment. Columns with the same letter or an asterisk within the same genotype are not significantly different.

In the presence of ethylene, ETHYLENE-INSENSITIVE2 (EIN2) activates a transcription factor (TF) cascade, including ETHYLENE INSENSITIVE3 (EIN3), and its homologs EIN3-like (EIL1/2/3) (Alonso and Stepanova, 2004). *EIL1* is the homolog most closely related to *EIN3* (Chao *et al.*, 1997) and, in accordance with this, the *ein3eil1* double-mutant shows an almost complete insensitivity to ethylene (Alonso *et al.*, 2003). For this reason, seedlings of *ein3eil1* mutant were analyzed in the presence or absence of ACC and with or without IBA, at the concentrations selected for the WT. The hypocotyl length remained unchanged under the application of ACC and/or IBA in comparison with the HF treatment (Fig. 3A), in contrast with the reduction that occurred in the WT, and in accordance with the inability of the mutant to perceive the ACC-derived ethylene and to inhibit hypocotyl elongation. The data for AR density confirmed the insensitivity of *ein3eil1* to the endogenous and exogenous (ACC-derived) ethylene, because the AR response was higher than in the WT under HF and with ACC alone (2- and 3.4-fold, respectively) and there were no significant differences between the two, which was in contrast with the significant reduction occurring with ACC in the WT (Fig. 3B). When IBA was applied alone, significant increases in AR density occurred in both the mutant and the WT (6-fold and 18-fold, respectively), in accordance with the persistent auxin-sensitivity of the mutant (Růžička *et al.*, 2007), but the AR density was significantly lower than in the WT (Fig. 3B). Moreover, the IBA+ACC treatment did not change the AR response in the mutant, showing that its ethylene-insensitivity negatively affected the AR promotion that was activated in the WT (Fig. 3B). Taken together, the response of this mutant shows that the ACC effect occurs through the EIN3EIL1 network, and that at least part of the IBA effect is mediated through this network.

### *The* wei2wei7 *AR response suggests that ACC inhibits the α- and β-anthranilate synthase genes involved in IAA biosynthesis*

**Fig. 4.**
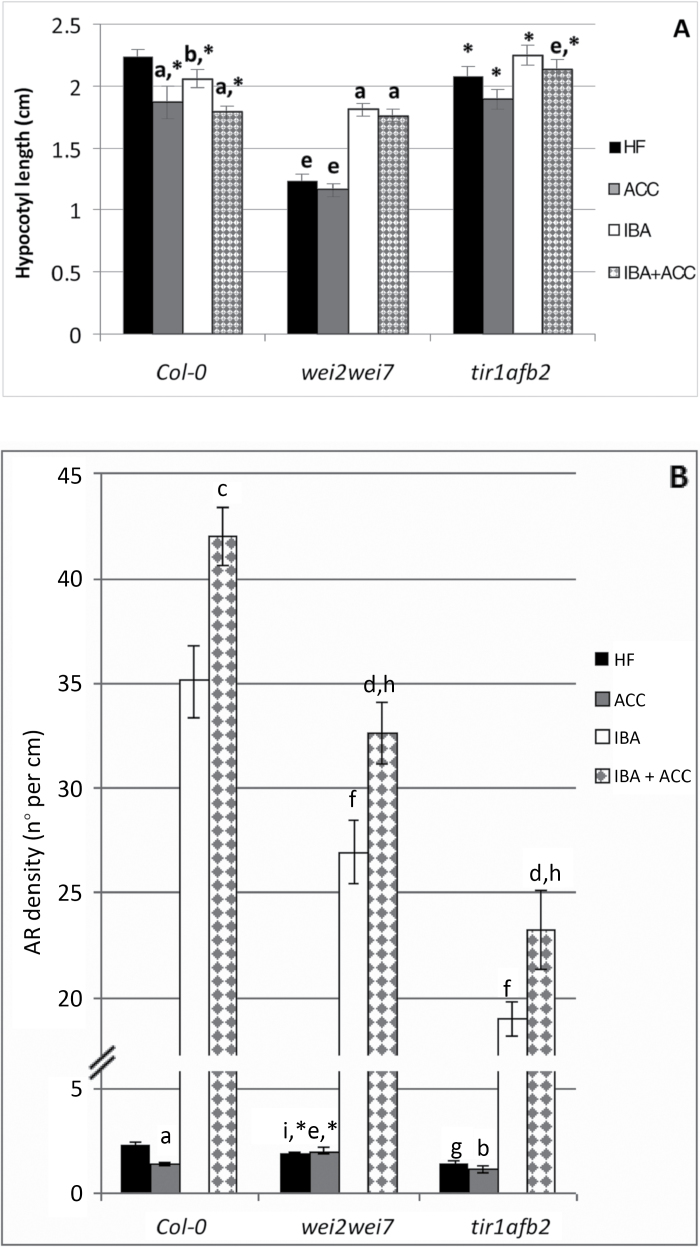
AR formation from hypocotyls of *Arabidopsis thaliana* seedlings of the Col-0 ecotype (WT), and of the *wei2wei7* and *tir1afb2* double-mutants at the end of *in vitro* growth (22 DAS) under continuous darkness either without hormones (HF), or with ACC (0.1 µM) and/or IBA (10 µM). (A) Mean hypocotyl length (±SE). (B), AR density, i.e. AR number per cm of hypocotyl, expressed as the mean value (±SE). *n*=30. a, *P*<0.01 difference with respect to HF within the same genotype; b, *P*<0.05 difference with respect to HF within the same genotype; c, *P*<0.01 difference with respect to IBA within the same genotype; d, *P*<0.05 difference with respect to IBA within the same genotype; e, f, g, h, *P*<0.01 difference with respect to the WT within the same treatment; i, *P*<0.05 difference with respect to the WT within the same treatment. Columns with the same letter or an asterisk within the same genotype are not significantly different.

To understand whether ethylene had an effect on IAA synthesis during the AR process, seedlings of the *wei2wei7* mutant, blocked at the level of the genes encoding the α and β subunits of anthranilate synthase that is involved in IAA-biosynthesis (Stepanova *et al.*, 2005), were treated with or without 0.1 μM ACC, and/or 10 μM IBA. The mutant showed a hypocotyl shorter than the WT. However, it reached a length similar to the WT in the presence of IBA (Fig. 4A), possibly because an IBA conversion into IAA compensated for the low endogenous IAA levels resulting from the mutation (Stepanova *et al.*, 2005). Moreover, ACC alone did not affect hypocotyl elongation in the mutant in comparison with HF, and did not cause any change in elongation when applied together with IBA in comparison with IBA alone (Fig. 4A). AR density was significantly reduced in the *wei2wei7* mutant in comparison with the WT under HF and, in contrast to the WT, ACC did not negatively affect the AR response (Fig. 4B). Taken together, the results from the mutant support the view that ethylene reduced AR formation in the WT by negatively affecting α- and β-anthranilate synthase gene activity. In the presence of exogenous IBA, *wei2wei7* produced many times more ARs than under HF (Fig. 4B), demonstrating the sustained presence in the mutant of IBA perception and conversion into IAA, as in the WT, even if AR production remained significantly lower than in the latter (*P<0.01*). Moreover, ACC significantly increased AR number in the presence of IBA, as in the WT, but at a significantly lower level (*P*<0.01; Fig. 4B). Taken together, these results suggest that the promotion of AR formation by IBA+ACC was independent of the IAA biosynthesis mediated by anthranilate synthase.

### ACC affects the AR process in the absence of auxin signaling mediated by TIR1-AFB2.

The TIR1/AFB (AUXIN SIGNALING F-BOX) proteins form the complexes responsible for the targeted degradation of the Aux/IAA repressors of the AUXIN RESPONSE FACTORS (ARFs), thus relieving ARF repression and allowing auxin-responsive gene transcription (Simon and Petrášek, 2011). TIR1 and AFB2 are the dominant auxin receptors in the PR, and the *tir1afb2* mutant is more resistant to auxin than *tir1* alone (Parry *et al.*, 2009). For this reason seedlings of the *tir1afb2* mutant were used in the present research. They showed a hypocotyl length comparable to the WT under HF. This length was not significantly affected by IBA, with or without ACC, and was significantly higher than in the WT under IBA+ACC (Fig. 4A), in accordance with the very high (but not complete) auxin-resistance of this mutant (Parry *et al.*, 2009). As expected, the AR response of the mutant was very low under the HF treatment, i.e. 1.7-fold lower than in the WT (*P*<0.01) and ACC reduced it significantly, as in the WT (Fig. 4B). In the presence of IBA alone, the AR density in *tir1afb2* was about 13-fold higher than under HF, even if the mean value remained significantly lower than in the WT, which supports a weaker induction activity by IBA in the mutant, in accordance with a similar effect on lateral root formation in *tir1* (Strader *et al.*, 2008). The addition of ACC with IBA caused a weak, but significant, increase in AR density in *tir1afb2* (Fig. 4B), suggesting that the mechanism by which ACC combined with IBA enhances AR formation in the WT remains effective in the mutant. In accordance with this, the mutation affected the steady-state levels of free IAA and IBA in the hypocotyls of the IBA+ACC-treated seedlings in a similar way to the WT, although to a lower extent (Table 1).

**Table 1.** Mean values (±SE) of IAA and IBA levels (ng g^−1^ FW) in hypocotyls with ARs excised from Col-0, *ein3eil1*, and *tir1afb2* seedlings at 22 DAS of growth under darkness either in the absence of exogenous hormones (HF), or in the presence of 0.1 μM ACC (ACC), or 10 μM IBA (IBA), or 0.1 μM ACC plus 10 μM IBA (IBA+ACC). Different lower-case letters indicate significant differences within the same genotype and auxin type, whereas no letter or an asterisk indicates no significant difference. Different upper-case letters indicate significant differences between genotypes within the same treatment and auxin type, whereas no letter indicates the absence of significant differences. *n*=3 replicates per genotype and treatment.

Treatments	Col-0	*ein3eil1*	*tir1afb2*
IAA (ng g^−1^ FW)			
HF	131.36 ± 3.83	191.31 ± 3.83 ^A^	141.50 ± 4.10
ACC	201.80 ± 8.44 ^a^	197.99 ± 6.07	196.10 ± 4.11 ^a^
IBA	227.30 ± 4.55 ^b^	234.84 ± 4.70 ^f,^*	144.06 ± 6.03 ^A^
IBA+ACC	310.03 ± 6.20 ^c,A^	212.14 ± 4.25 *	210.70 ± 12.26 ^a,c^
IBA (ng g^−1^ FW)			
HF	10.18 ± 0.73	10.12 ± 0.51	8.08 ± 0.85
ACC	23.74 ± 1.13 ^a,A^	9.44 ± 0.47	11.37 ± 0.97
IBA	33.28 ± 1.66 ^d^	28.09 ± 1.90 ^d,B,^*	12.07 ± 0.43 ^A^
IBA+ACC	28.58 ± 0.59 ^e^	29.00 ± 2.19*	9.33 ± 1.17 ^A^

^a^ *P*<0.0001 difference with respect to HF treatment; ^b^ *P*<0.0001 difference with respect to HF, and *P*<0.05 difference with respect to ACC alone; ^c^ *P*<0.0001 difference with respect to IBA alone; ^d^ *P*<0.0001 difference with respect to HF and ACC alone; ^e^ *P*<0.0001 difference with respect to HF, and *P*<0.05 difference with respect to IBA alone and ACC alone; ^f^ *P*<0.001 difference with respect to HF, and *P*<0.01 difference with respect to ACC alone; ^A^ *P*<0.0001 difference with respect to the other genotypes; ^B^ *P*<0.05 difference with respect to Col-0.

### ACC affects the steady-state levels of IAA and IBA in the hypocotyls that form ARs

The steady-state levels of endogenous IAA and IBA were determined in the AR-forming hypocotyls. In the first instance, the quantification of the two auxins was carried out in the WT under HF and ACC alone (0.1 μM) at 14 DAS, because at this time the AR response in these treatments had already reached a mean AR density comparable to that at 22 DAS (Fig. 2B), i.e. 1.6(±0.2) and 1.1(±0.2), respectively. The IAA content was about 14-fold higher than the IBA content, in accordance with previous data in seedlings of the same species under different conditions (Ludwig-Müller *et al.*, 1993), and the presence of ACC did not cause any significant changes in either the IAA or IBA levels (Supplementary Table S1).

At 22 DAS, the levels of the two auxins were determined in the WT, *ein3eil1*, and *tir1afb2* mutants under all treatments (Table 1). In comparison with 14 DAS, at 22 DAS no significant changes in IAA and IBA levels occurred in the WT under HF, whereas significant increases occurred with ACC (*P*<0.01 for IAA, and *P*<0.001 for IBA), leading to IAA and IBA levels significantly higher than under HF (Table 1 and Supplementary Table S1). As expected, exogenous IBA greatly enhanced IAA and IBA levels in comparison with HF and, when combined with ACC, caused a further, and highly significant, enhancement in IAA levels, but a highly significant reduction in IBA levels (Table 1), suggesting that the addition of ACC to exogenous IBA had favoured IBA-to-IAA conversion.

In the HF-grown *ein3eil1* hypocotyls, IAA levels significantly increased in comparison with the WT (*P*<0.0001; Table 1), as a possible consequence of their higher number of ARs (Fig. 3B), which provide additional sites of auxin biosynthesis (Della Rovere *et al.*, 2016). In accordance with the ethylene-insensitivity of this mutant, no significant changes in IAA and IBA levels were caused by ACC alone in comparison with HF, and by IBA+ACC in comparison with IBA alone (Table 1). In contrast, both IAA and IBA greatly increased under IBA alone in comparison with HF and ACC alone (Table 1), altogether suggesting that the ACC effect in modulating IAA and IBA levels was dependent on the EIN3EIL1 transcriptional network.

The hypocotyls of *tir1afb2* seedlings grown under HF and IBA alone showed low IAA levels that were comparable between the two, and with the WT under HF (no significant difference), but that were greatly reduced in comparison with the WT under exogenous IBA (Table 1). IBA levels were low and similar under all treatments, but highly significantly reduced under exogenous IBA when compared with the WT (Table 1). These results are in accordance with the high, but not complete, auxin-insensitivity caused by the *tir1afb2* mutation (Parry *et al.*, 2009) and with the low AR response of *tir1afb2* under HF (Fig. 4B), but they do not explain the conspicuous rise in the AR response under IBA (Fig. 4B). It is possible that the IBA-to-IAA conversion machinery remained active in the mutant and allowed exogenous IBA to elevate IAA levels and thus give rise to AR formation, but it is also possible that IBA *per se*, rather than its conversion to IAA, stimulated AR formation. When combined with IBA, ACC significantly enhanced IAA levels and reduced, even if not significantly, IBA levels (Table 1), suggesting that an ACC-enhancement of IBA-to-IAA conversion was also active in the mutant to support the observed increase in the AR response in comparison with IBA alone (Fig.4B), and that ethylene functioned independently of TIR1/AFB2 IAA signaling.

### *The* ech2ibr10 *AR response supports the hypothesis that ACC-enhanced IBA-to-IAA conversion is needed for enhancing AR formation*

Several enzymes appear to be specific for IBA-to-IAA conversion, and mutations in genes encoding these enzymes confer IBA resistance without altering the IAA response (Strader and Bartel, 2011). Candidates include the dehydrogenase/reductase INDOLE-3-BUTYRIC ACID RESPONSE1 (IBR1), the enoyl-CoA hydratase IBR10, and ENOYL-COA HYDRATASE2 (ECH2). All *ibr* mutants exhibit defects in IBA responses (Strader and Bartel, 2011), and the phenotype of *ech2-1* is synergistic with those of the *ibr* mutants, *ibr10* in particular (Strader *et al.*, 2011).

To understand whether the AR increase caused by ACC, when applied with IBA, was due to a promotion of IBA-to-IAA conversion, the AR response of seedlings of the *ech2ibr10* mutant was analyzed.

**Fig. 5.**
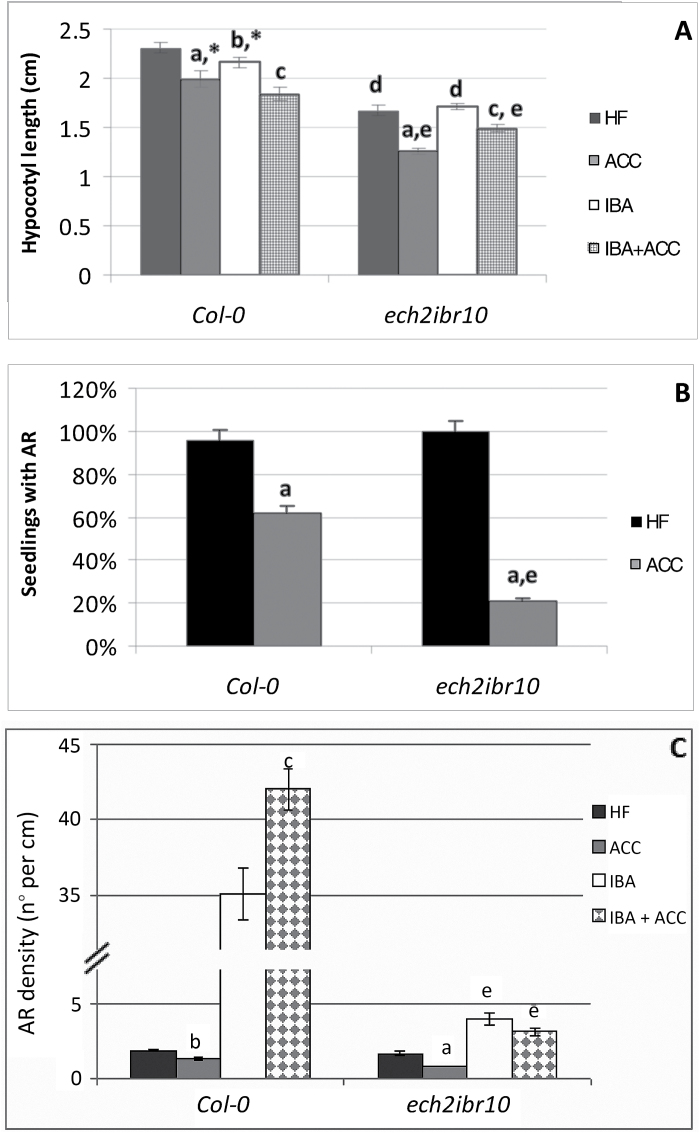
AR formation from hypocotyls of *Arabidopsis thaliana* seedlings of the Col-0 ecotype (WT) and the *ech2ibr10* mutant at the end of *in vitro* growth (22 DAS) under darkness with or without ACC (0.1 µM) and/or IBA (10 µM). (A) Mean hypocotyl length (±SE). (B) Percentage of seedlings with ARs under HF and ACC. (C) AR density, i.e. AR number per cm of hypocotyl, expressed as the mean value (±SE). *n*=30. a, *P*<0.01 difference with respect to HF within the same genotype; b, *P*<0.05 difference with respect to HF within the same genotype; c, *P*<0.01 difference with respect to IBA alone within the same genotype; d, e, *P*<0.01 difference with respect to the WT, within the same treatment. Columns with the same letter or an asterisk within the same genotype are not significantly different. Columns with no letters in different genotypes are not significantly different.

The hypocotyls of the mutant were significantly shorter than those of the WT under HF and their length was reduced by ACC, alone or combined with IBA, with a trend similar to that of the WT. In accordance with the possible absence in the mutant of an active IBA-to-IAA conversion, the hypocotyl length under IBA alone was not reduced in comparison with HF (Fig. 5A). The percentage of seedlings with ARs and the AR density were reduced by ACC in comparison with HF at a significantly higher level in the mutant than in the WT (Fig. 5B-C), supporting an effect of ACC on the conversion of endogenous IBA into IAA in the WT, which would be useful for AR formation. In the presence of exogenous IBA, there was a 2.4-fold increase in the AR response of *ech2ibr10*, suggesting a possible IBA-promotion of AR formation independent of IBA-to-IAA conversion in this mutant (Fig. 5C), as in *tir1afb2* (Fig. 4B), although it was 4.8-fold lower (compare Figs 4B and 5B). Differently from the WT, ACC did not cause significant variations (Fig. 5C), showing that exogenous IBA, when applied with ACC, acted particularly on the AR process by conversion into IAA in the WT, whereas this did not occur in the mutant because of the inactivation of the conversion machinery.

### The expression of IAA biosynthetic genes during AR formation is reduced by exogenous IBA combined with ACC

**Fig. 6.**
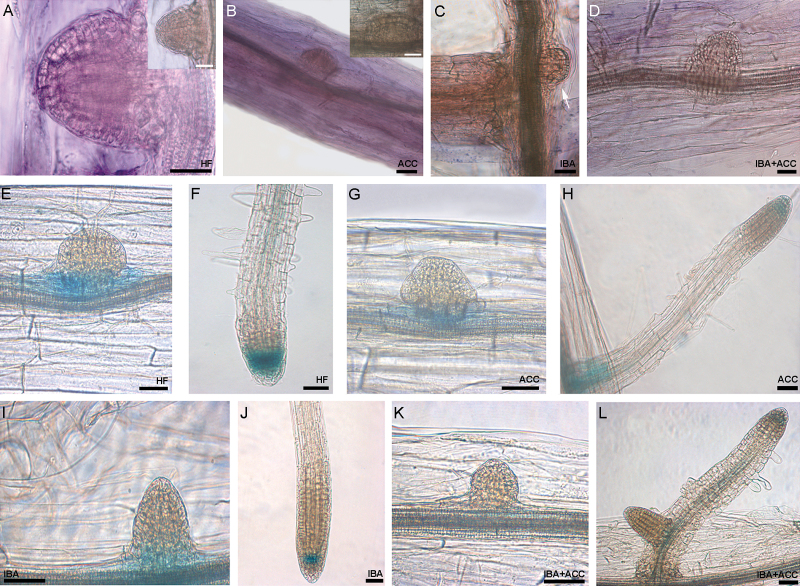
Whole-mount RNA *in situ* hybridizations of *YUC6* (A–D) in Col-0 seedlings, and histochemical GUS analysis in the *ASA1::GUS* transgenic line (Col-0 background) (E–L) at 22 DAS of growth in the presence/absence of IBA and/or ACC. (**A**) Hybridization signal of the antisense probe in a domed ARP under HF. (B, C) Weak hybridization signal in a domed ARP under ACC alone (B), and under IBA alone (C, arrow). (D) Almost absent hybridization signal in an ARP under IBA+ACC. Insets in (A) and (B) show the absence of hybridization signal in domed ARPs of the samples treated with the sense probe. (E, F) *ASA1* expression at the base of an early domed ARP (E) and strong expression in the apex of a mature AR (F) (HF treatment). (G, H) Weak *ASA1* expression under ACC alone at the base of an early domed ARP (G), and at the base and, faintly, in the apex of a mature AR (H). (I, J) *ASA1* expression at the base of a domed ARP (I) and at the tip of a mature AR (J) (IBA alone). (K, L) Highly reduced gene expression at the base of a domed ARP (K), and at the tip of a mature AR (L) under IBA+ACC. Scale bars are 40 μm.

A whole-mount RNA *in situ* hybridization analysis of *YUC6* was carried out at 22 DAS in WT seedlings in order to understand the effect of 0.1 μM ACC on the expression of this IAA biosynthetic gene, which is known to be positively involved in Arabidopsis AR formation (Della Rovere *et al.*, 2016). In the presence of either ACC alone or IBA alone, the expression sites of the gene in the hypocotyl and in the AR stages were the same as under HF, as exemplified for the early domed ARPs in Fig. 6A–C. However, in comparison with HF (Fig. 6A), the signal was weaker with ACC (Fig. 6B) and much weaker with IBA (Fig. 6C). The signal was further reduced with IBA+ACC at all the AR stages, as exemplified for the early domed ARP stage in Fig. 6D in comparison with Fig. 6A–C. These results indirectly support an ACC inhibitory effect on YUC6-mediated IAA biosynthesis, and a reduction in such biosynthesis caused by exogenous IBA, and by IBA+ ACC in particular.

In addition, seedlings of *ASA1::GUS* and *ASB::GUS* transgenic lines expressing GUS under the control of the promoters of the α- and β-anthranilate synthase genes, which are involved in IAA biosynthesis upstream of the *YUC* genes (Brumos *et al.*, 2014), were also analyzed.

At 22 DAS under HF, *ASA1* expression was present from the AR formative divisions to all further AR stages, e.g. in the domed ARPs (Fig. 6E) and was strongly present in the apex of mature ARs (Fig. 6F). In the presence of ACC alone, the signal was present in the same sites as under HF, but it was weaker (compare Fig. 6G, H and E, F), reinforcing the interpretation of the results obtained with the *wei2wei7* mutant (Fig. 4B). Under exogenous IBA alone, a weak reduction of *ASA1* expression occurred in comparison with HF, but this reduction was less than that observed under ACC alone (Fig. 6I, J, and E–H). In the presence of IBA+ACC, a strong reduction of the *ASA1* signal occurred (Fig. 6K, L). The *ASB* expression pattern was similar to that of *ASA1* under HF and IBA alone (Supplementary Fig. S5A–E), whereas ACC caused a weaker reduction in the signal in comparison with *ASA1,* both applied alone and combined with IBA (Supplementary Fig. S5F–J), suggesting that ACC specifically reduced the transcriptional induction of the α-anthranilate synthase isoform (Fig. 6G, H, K, L).

### *ACC enhances the signal of the auxin* DR5::GUS *reporter system in forming ARs in the presence of exogenous IBA, and reduces it in its absence*

**Fig. 7.**
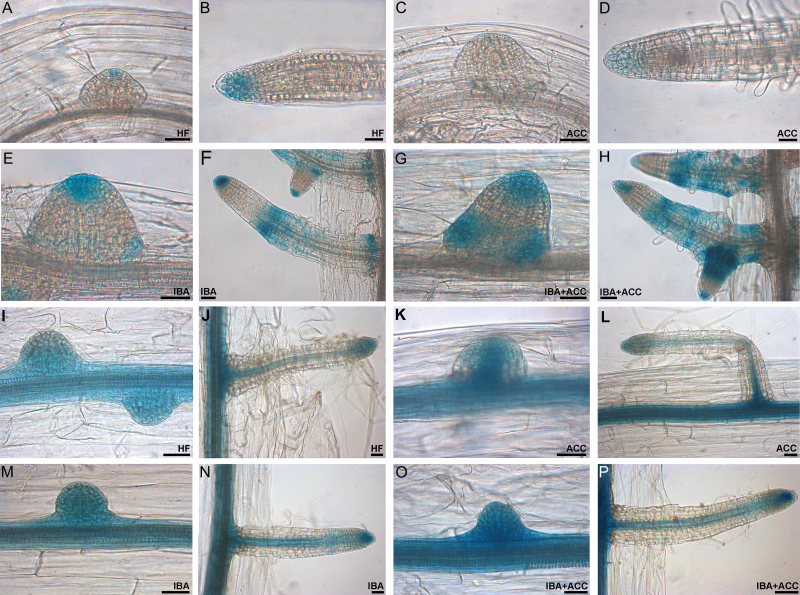
Histochemical GUS staining analysis of *DR5::GUS* (A–H) and *PIN1::GUS* (I–P) seedlings at 22 DAS of growth under darkness, either without hormones (HF), or with ACC (0.1 µM) and/or IBA (10 µM) (Col-0 background). (A, B) *DR5::GUS* staining at the tip of a domed ARP (A) and of a mature AR (B) (HF). (C, D) Very weak IAA gene expression signal in a few apical cells of a domed ARP (C) and in the apex of a protruded AR (D) (ACC alone). (E, F) Strong *DR5::GUS* staining at the tip of a domed ARP and weakly at the base (E), and in the same sites but also in the elongation/differentiation zone of mature ARs (F) (IBA alone). (G, H) Enhanced *DR5::GUS* signal at the tip and base of a domed ARP (G), and at the tip, base, and more widely in the mature ARs (H) (IBA+ACC). (I, J) *PIN1* expression pattern in early ARPs and in the hypocotyl vasculature at their bases (I), and at the AR tip and along the hypocotyl and AR vasculature (J) (HF). (K, L) *PIN1* expression pattern in the vasculature of the hypocotyl and in an adjacent domed ARP (K), and in the hypocotyl and AR vasculature, and at the tip of a mature AR (L) (ACC alone). (M–P) Very intense *PIN1* expression in early domed ARPs (M, O) and mature ARs (N, P) under IBA alone (M, N), and IBA+ACC (O, P) (same expression sites as I–L). Scale bars are 30 μm.

The *DR5::GUS* line, a well-known reporter of auxin-induced gene expression, was used to support the results concerning ACC effects on the localization of *YUC6* and *ASA1* gene expression. Under HF, the *DR5::GUS* construct was expressed at the tip of early domed ARPs (Fig. 7A) and in the quiescent centre, flanking initials, and cap cells of the apex of mature ARs (Fig. 7B, Della Rovere *et al.*, 2013). In the presence of ACC, the *DR5::GUS* signal was still present, but weaker than under HF (compare Fig.7C, D and A, B), in accordance with the ACC effect on the expression of *YUC6* and *ASA1* genes (Fig. 6B and G, H). Exogenous IBA enhanced the intensity and localization of GUS staining during the entire AR process in comparison with both the HF and ACC-alone treatments, marking the tip and base of ARPs (Fig. 7E) and ARs (Fig. 7F), but also the elongation/differentiation zone of the ARs (Fig. 7F). In seedlings grown with IBA+ACC the staining was stronger than under IBA alone at all AR stages, and was observed not only in the same sites in the ARPs and ARs (Fig. 7G, H) but also in a wide part of the AR primary body (compare Fig. 7H and F). Altogether, the results showed that ACC reduced the auxin-induced gene expression in the absence of IBA, but caused a reinforced and more extended signal in its presence.

### The activity of the promoter of the IAA cellular-efflux-carrier PIN1 is not affected by ACC during the AR process

The possibility that the ACC-derived ethylene reduced AR formation by affecting IAA cellular efflux was investigated by analyzing the expression of *PIN1*, using the *PIN1::GUS* line. The activity of the promoter of *PIN1* was investigated because it is known that it is involved in AR formation from the hypocotyls of intact Arabidopsis seedlings grown under darkness (Della Rovere *et al.*, 2013), and because the *pin1* mutation has been reported to negatively affect AR formation in hypocotyls of de-rooted seedlings of Arabidopsis grown in low-light (Sukumar *et al.*, 2013).

Under all treatments, *PIN1* expression was observed from early divisions to doming ARPs (Fig. 7I, K, M, O) and continued up to the AR stage, marking the AR conjunction with the hypocotyl and the AR vasculature and apex (Fig. 7J, L, N, P). A weak increase in expression levels occurred with IBA, alone or combined with ACC, in particular at the early ARP-stages (Fig. 7M, O), in accordance with the auxin-inducibility of this gene (Vieten *et al.*, 2005). However, the absence of differences caused by ACC in *PIN1* expression suggests that either the gene is post-transcriptionally controlled or that ethylene effects on AR formation do not depend on PIN1-mediated IAA cellular efflux.

### IBA efflux by ABCG transporters is not affected by ACC during the AR process

Two members of the PLEIOTROPIC DRUG RESISTENCE subclade of the ABCG family, namely ABCG36 and ABCG37, are known to be involved in IBA efflux in the PR cells of Arabidopsis, and the *abcg36abcg37* double-mutant shows an IBA-hyperaccumulating phenotype (Strader and Bartel, 2011). Thus, the AR response of *abcg36abcg37* was investigated in order to obtain information about possible ACC effects on IBA efflux.

**Fig. 8.**
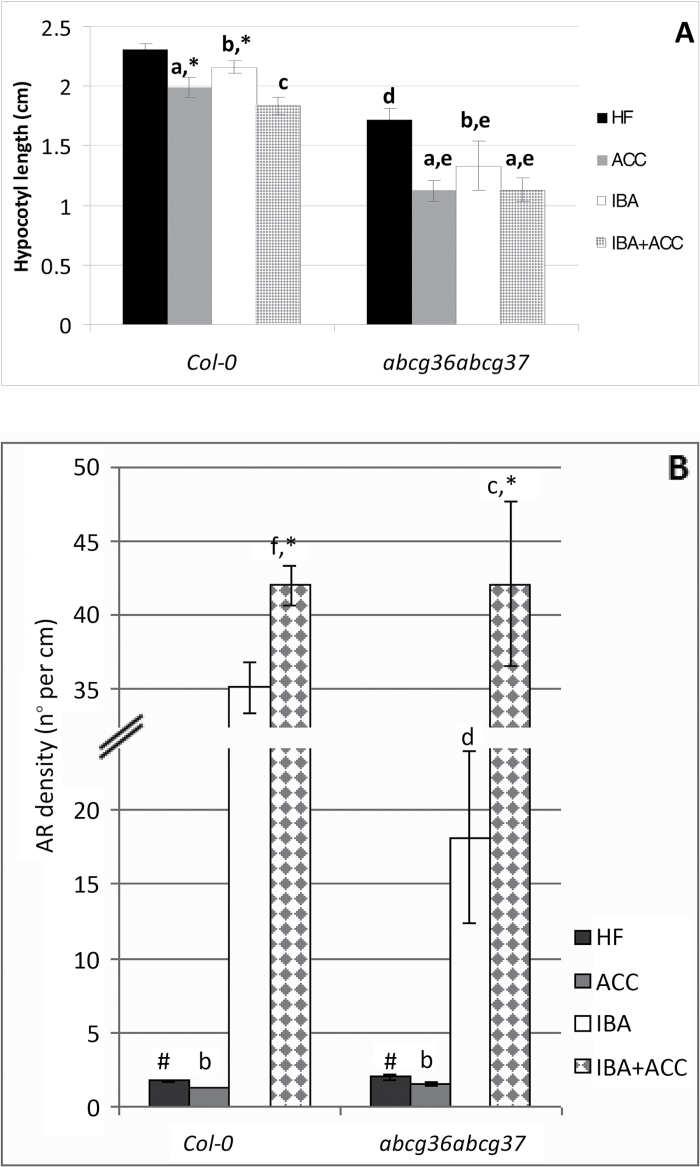
AR formation from hypocotyls of *Arabidopsis thaliana* seedlings of the Col-0 ecotype (WT) and the *abcg36abcg37* double-mutant at the end of *in vitro* growth (22 DAS) under darkness with or without ACC (0.1 µM) and/or IBA (10 µM). (A) Mean hypocotyl length (±SE). (B) AR density, i.e. AR number per cm of hypocotyl, expressed as the mean value (±SE). *n*=30. a, *P*<0.01 difference with respect to HF within the same genotype; b, *P*<0.05 difference with respect to HF within the same genotype; c, *P*<0.01 difference with respect to IBA within the same genotype; d, e, *P*<0.01 difference with respect to the WT within the same treatment; f, *P*<0.05 difference with respect to IBA alone within the same genotype. Columns with the same letter or symbol are not significantly different.

The hypocotyls of the *abcg36abcg37* double-mutant were significantly shorter than in the WT under all growth conditions, but the trend of length-reduction caused by ACC, with or without IBA, was the same as in the WT (Fig. 8A). Under HF, the mutant showed an AR density similar to the WT, and a similar decrease caused by ACC (Fig. 8B), indicating similar ACC effects on AR production. In the presence of IBA alone, the AR density was enhanced by about 10-fold, but it remained significantly lower than in the WT (Fig. 8B), as expected by the mutation. However, IBA+ACC significantly increased the AR response up to values similar to the WT (Fig. 8B), suggesting that this increase was also caused by an ACC-enhanced IBA-to-IAA conversion in the mutant. Taken together, these results exclude the possibility that the ethylene action on the AR process depends on the cellular IBA efflux by the ABCG36 and ABCG37 transporters.

### IAA influx by the AUX1 and LAX3 carriers is necessary for ACC action during the AR process

Based on the roles of the IAA influx carriers AUX1 and LAX3 on the AR process (Della Rovere *et al*., 2013, 2015), the effect of ACC on these two carriers was also monitored by analyzing the response of the *lax3aux1* mutant and the expression patterns of the promoters of the two genes, using the *AUX1::GUS* and *LAX3::GUS* lines.

**Fig. 9.**
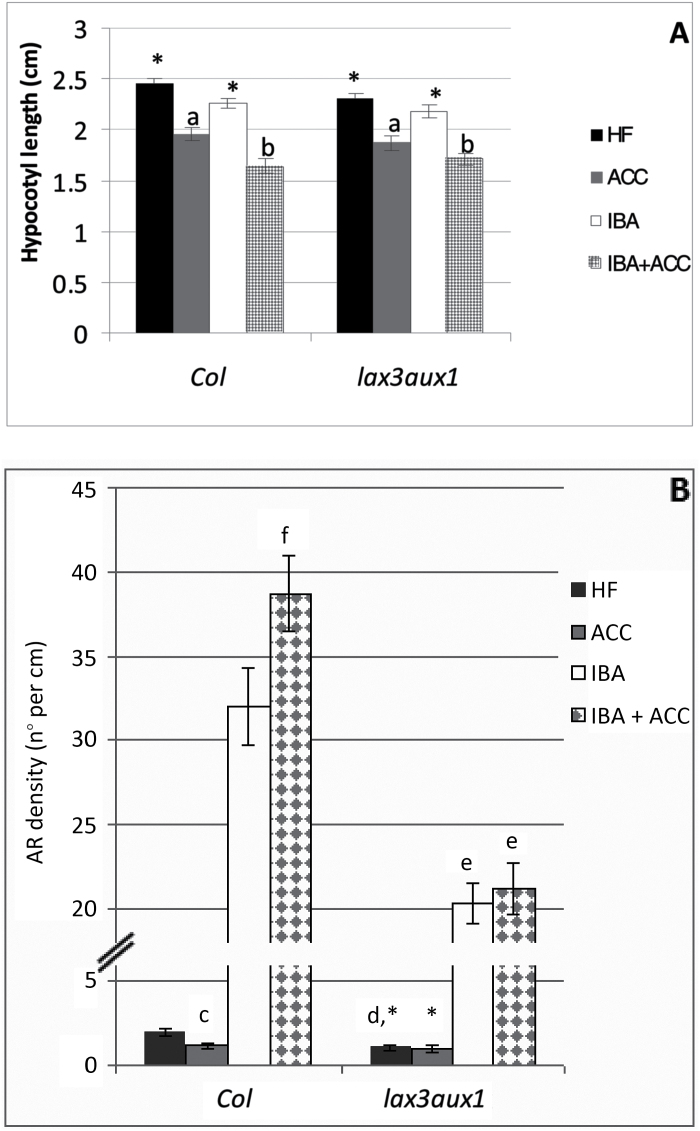
AR formation from hypocotyls of *Arabidopsis thaliana* seedlings of the Col ecotype (WT) and the *lax3aux1* double-mutant at the end of *in vitro* growth (22 DAS) under darkness with or without ACC (0.1 µM) and/or IBA (10 µM). (A) Mean hypocotyl length (±SE). (B) AR density, i.e. AR number per cm of hypocotyl, expressed as the mean value (±SE). *n*=30. a, *P*<0.01 difference with respect to HF within the same genotype; b, *P*<0.01 difference with respect to HF and IBA alone within the same genotype; c, *P*<0.05 difference with respect to HF within the same genotype; d, e, *P*<0.01 difference with respect to the WT within the same treatment; f, *P*<0.01 difference with respect to IBA alone within the same genotype. Columns with the same letter or an asterisk are not significantly different.

The hypocotyl length in *lax3aux1* seedlings was similar to the WT under all treatments (Fig. 9A). However, under HF the hypocotyl of the mutant produced far fewer ARs than the WT, which accords with the known positive involvement of both carriers in AR formation (Della Rovere *et al.*, 2015). However, differently from the WT, no further inhibition in AR formation was caused by ACC in the mutant (Fig. 9B). In addition, in the presence of IBA the AR density of *lax3aux1* was significantly lower than in the WT (*P<0.01*), and ACC did not change it significantly (Fig. 9B).

**Fig. 10.**
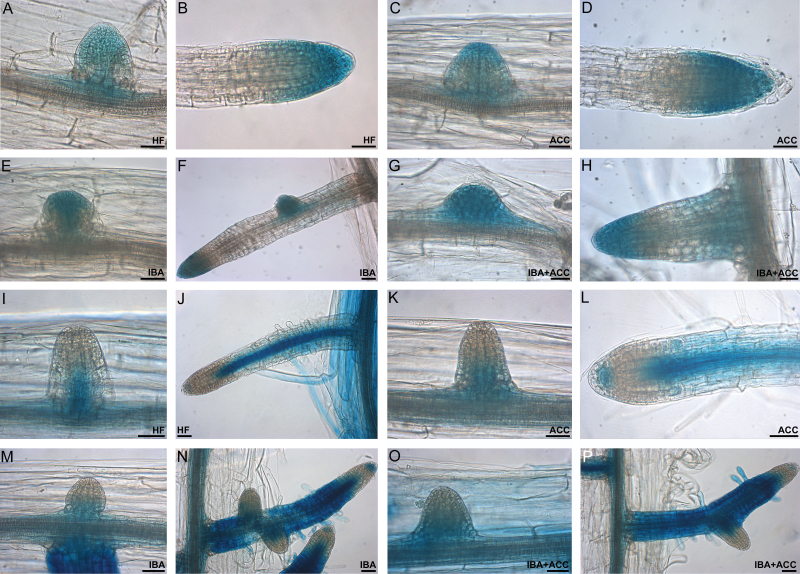
Histochemical GUS staining analysis of *AUX1::GUS* (A–H) and *LAX3::GUS* (I–P) seedlings at 22 DAS of growth under darkness in the presence/absence of IBA and/or ACC (Col-0 background). (A–D) *AUX1* expression in domed ARPs (A, C) and mature AR tips (B, D) under HF (A, B) and ACC (C, D). (E–H) *AUX1* expression in domed ARPs (E, G) and ARs (F, H) under IBA (E, F) and IBA+ACC (G, H), showing no relevant differences in expression sites and intensity with (A–D). (I, J) *LAX3* expression at the base of a domed ARP (I) and along the vasculature and in a few apical cells in mature ARs (J) (HF). (K, L) *LAX3* expression in domed ARPs (K), and mature ARs (L), showing the same pattern as under HF (ACC). (M–P) Stronger *LAX3* expression in the same AR stages as (I–L), in the presence of exogenous IBA alone (M, N) or combined with ACC (O, P). Scale bars are 30 μm.

Histochemical analysis of *AUX1::GUS* seedlings showed that the exogenous IBA caused no change in the expression signal of *AUX1* at any AR stage in comparison with the HF treatment (Fig. 10A, B and E, F), in accordance with the notion that AUX1 recognizes endogenous IAA but not IBA (Liu *et al.*, 2012). ACC (+/– IBA) did not change the expression pattern of the gene (Fig.10C, D, G, H). In the presence of ACC alone, analysis of *LAX3::GUS* seedlings showed that the *LAX3* signal was similar in intensity and tissue localization to HF (compare Fig. 10I, J and K, L). A stronger signal was caused by exogenous IBA, both alone and with ACC (Fig.10M, N and O, P), in accordance with the known IBA-inducibility of this gene (Liu *et al.*, 2012). The signal occurred in the same cellular sites in HF and ACC alone (Fig.10I–L), but it was too intense to allow detection of differences between IBA and IBA+ACC (Fig. 10M–P). Collectively, the results suggest that IAA cellular influx by AUX1 and LAX3 is necessary for ethylene action, but the activity of the coding genes seems post-transcriptionally controlled.

## Discussion

The results show that crosstalk between IAA, IBA, and ethylene is essential for AR formation in the etiolated seedlings of Arabidopsis. Ethylene, produced by its precursor ACC, modulates the AR response by negatively affecting IAA synthesis but by positively enhancing the conversion of the IAA-precursor IBA into active free IAA, and its activity depends on the IAA-influx carriers AUX1 and LAX3. Moreover, exogenous IBA is the hormone that promotes AR formation in the etiolated Arabidopsis seedlings, and part of the AR promotion by IBA is mediated by ethylene through the EIN3EIL1 network, as shown by the response of the *ein3eil1* double-mutant. Another part of the action of IBA seems to be ethylene-independent. Whether IBA itself is able to induce responses independently of IAA remains to be resolved in Arabidopsis, as in other plants (Sauer *et al.*, 2013); however, the present data seem to support this possibility for AR formation, giving a new insight to the debate about IBA functioning *per se*. Thus, the AR density of the IAA-resistant *tir1afb2* mutant and (albeit to a lower extent) that of the *ech2ibr10* mutant (which is blocked in IBA-to-IAA conversion) increases in the presence of IBA alone in comparison with the HF treatment. By contrast, ethylene enhances AR formation, when combined with IBA, by favouring its conversion into IAA, i.e. favouring the usual route of IBA indirect activity (Strader and Bartel, 2011; Sauer *et al.*, 2013). This is shown by the AR response of *ech2ibr10* in the presence of IBA+ACC, which is not significantly different from that of IBA alone, and by the enhanced levels of endogenous IAA and reduced levels of IBA in the WT at the end of the growth period under the same treatment. The ethylene-favoured IBA-to-IAA conversion as the source of the IAA necessary for sustaining AR formation might result in an inhibition of IAA biosynthesis because it is not necessary to the process. The analyses of the expression of *WEI2* (*ASA1*) and *WEI7* (*ASB*), which are involved in the ethylene-induced IAA biosynthetic pathway (Stepanova *et al.*, 2005), and of the transcription of *YUC6* of the same Trp-dependent pathway (Brumos *et al.*, 2014), together with the *wei2wei7* AR response, support this hypothesis.

The IBA conversion to IAA is also caused by ACC in the absence of exogenous IBA; however, AR formation is reduced in this case. The timing of IAA release from IBA seems to be important, because at 14 DAS the levels of the two endogenous hormones were not yet significantly different under ACC alone in comparison with the HF treatment. At 22 DAS, in contrast, IAA accumulated endogenously in the presence of ACC alone, but this occurred after the formation of the bulk of the ARs in this treatment (14 DAS), suggesting that during the last period of growth IAA was produced from IBA in order to induce an alternative morphogenic program to AR formation, e.g. xylogenesis. Xylogenesis consists of the ectopic formation of xylary elements and it is induced by auxin (Ricci *et al.*, 2016, and references therein) and by ethylene (Pesquet and Tuominen 2011), and occurs as an alternative or competitive program to AR formation in cuttings of numerous species (Faivre-Rampant *et al.*, 2003; Ricci *et al.*, 2016). Moreover, in etiolated Arabidopsis seedlings, the same auxin-reactivated pericycle cells can produce either ARs or xylary elements, with xylogenesis needing lower levels of auxin to be initiated (Della Rovere *et al.*, 2015). In agreement with this, a preliminary histological analysis showed that at 22 DAS xylogenesis was occasionally seen under IBA+ACC, but was greatly enhanced under ACC alone (MM Altamura, unpublished data).

In Arabidopsis, the ARs formed by the hypocotyl of etiolated seedlings need IAA cellular transport by AUX1, LAX3, and PIN1 (Della Rovere *et al*., 2013, 2015). In accordance with this, the observed response of the *lax3aux1* double-mutant demonstrates that an IAA influx by AUX1 and LAX3 in the target cells of the AR process, and in their derivative cells that are involved in building up the ARP, is necessary for ethylene action. It has also been demonstrated that AR formation in intact hypocotyls of etiolated seedlings of Arabidopsis is highly reduced in the *aux1* single-mutant, but not in the *lax3* one (Della Rovere *et al.*, 2015). The present data do not allow us to establish whether both the influx carriers are related to ethylene action; however, the absence of changes in the expression pattern of each gene in response to ACC causes us to hypothesize that both are post-transcriptionally controlled by the hormone.

In addition, the activity of the promoter of the IAA cellular-efflux-carrier *PIN1* is not affected by ethylene. Coupled with the inhibition of AR formation reported for de-rooted seedlings of Arabidopsis *pin1* (Sukumar *et al.*, 2013), the present results suggest that either this carrier is also post-transcriptionally controlled, or that other efflux transporters are activated by ethylene, e.g. the ATP-binding cassette B19, which is known to be involved in Arabidopsis AR formation (Sukumar *et al.*, 2013).

**Fig. 11.**
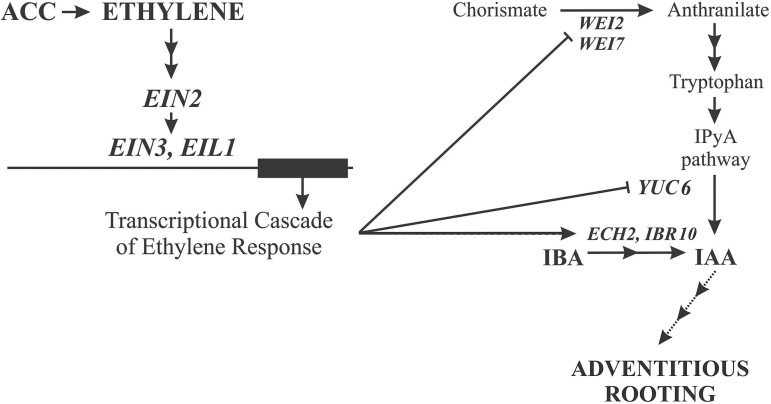
Model summarizing the main results regarding ethylene action on AR formation in etiolated Arabidopsis seedlings. ACC degradation by ACO gives rise to ethylene. In the presence of ethylene, EIN2 activates a TF cascade, including EIN3 and its most closely related homolog EIL1. The EIN3–EIL1 network activates a transcriptional cascade leading to the ethylene response. In the hypocotyl, the ethylene response involves the inhibition of the expression and activity of the WEI2 (ASA1) and WEI7 (ASB) isoforms of the anthranilate synthase that converts chorismate into anthranilate, an early step in the tryptophan-dependent indole-3-pyruvic acid (IPyA) pathway of IAA biosynthesis. Following this biosynthethic route, tryptofan is formed, and IPyA is in turn formed from tryptophan. IPyA is converted to IAA by YUC6 flavin monooxigenase. In addition, *YUC6* transcription is inhibited in response to ethylene, whereas the activities of the ENOYL-COA HYDRATASE IBR10 and the ENOYL-COA HYDRATASE2 (ECH2) of the β-oxidation of IBA, leading to its conversion into IAA, are stimulated. The IBA-derived IAA is the main source of auxin that leads to adventitious rooting through numerous successive steps (dotted arrows). Further details and references are in the text.

In conclusion, IBA serves as important regulator of IAA activity during AR formation in etiolated Arabidopsis seedlings, and ethylene mediates the regulating activity of IBA, according to the model summarized in Fig. 11. The ethylene–IBA crosstalk strengthens the importance of endogenous IBA in governing IAA levels within the seedling to support AR formation, and the necessity of a control by ethylene via IBA-to-IAA conversion. These results may help in understanding the mechanisms/compounds that regulate the homeostasis of the endogenous auxin pool, and thus be useful for improving rooting in microcuttings of recalcitrant species with economic value.

## Supplementary data

Supplementary data are available at *JXB* online.

Fig. S1. HPLC chromatogram for IAA and IBA determination (HF treatment).

Fig. S2. Percentage of Col-0 seed germination under all the treatments at 7 DAS, and effects of different ACC concentrations on the AR density in Col-0 seedlings at 22 DAS under darkness (replicate).

Fig. S3. AR density in Col-0*, ein3eil1*, *wei2wei7*, and *ech2ibr10* seedlings grown *in vitro* under darkness for 22 DAS, in the presence of HF, IBA, ACC, or IBA+ACC (replicate).

Fig. S4. Hypocotyl length and AR density in Col seedlings grown *in vitro* under darkness for 22 DAS, in the presence of HF, IBA, ACC, or IBA+ACC.

Fig. S5. Expression pattern of the β-anthranilate synthase gene during AR-formation under HF, IBA, ACC, or IBA+ACC.

Table S1. IAA and IBA levels in hypocotyls with ARs excised from Col-0 seedlings grown under HF or with ACC at 14 DAS.

Supplementary Data
