# Supplementary material for: Ethylene and auxin interaction in the control of adventitious rooting in Arabidopsis thaliana
Source: J Exp Bot. 2016 Nov 9;67(22):6445–58. doi: 10.1093/jxb/erw415 (PMC5181586; doi:10.1093/jxb/erw415)
Supplement: Supplementary Data [file supp_67_22_6445__index.html]

Ethylene and auxin interaction in the control of adventitious rooting in Arabidopsis thaliana — Ethylene and auxin interaction in the control of adventitious rooting in Arabidopsis thaliana — Supplementary Data 

# Ethylene and auxin interaction in the control of adventitious rooting in *Arabidopsis thaliana*

## Supplementary Data

Data files

- supplementary\_figures\_S1\_S5\_table\_S1.pdf - Supplementary Data
