# Supplementary material for: Ethylene and auxin interaction in the control of adventitious rooting in Arabidopsis thaliana
Source: J Exp Bot. 2016 Nov 9;67(22):6445–58. doi: 10.1093/jxb/erw415 (PMC5181586; doi:10.1093/jxb/erw415)
Supplement: Supplementary Data [file supp_erw415_supplementary_figures_S1_S5_table_S1.pdf]

Supplementary data of:

Ethylene and auxin interaction in the control of adventitious rooting in *Arabidopsis thaliana*

Veloccia A., Fattorini L., Della Rovere F., Sofo A., D'Angeli S., Betti C., Falasca G., and Altamura MM

5 Figures
1 Table

Supplementary Fig. 1 HPLC chromatogram for IAA and IBA determination (HF treatment).

Supplementary Fig. 2 Percentage of Col-0 seed germination under all the treatments at 7DAS, and effects of different ACC concentrations on the AR-density in Col-0 seedlings at 22DAS under darkness (replicate).

Supplementary Fig. 3 AR-density in Col-0, *ein3eil1*, *wei2wei7*, *ech2ibr10* seedlings, grown *in vitro* under darkness for 22DAS, in the presence of HF, IBA, ACC, and IBA+ACC (replicate).

Supplementary Fig. 4 Hypocotyl length and AR-density in Col seedlings grown *in vitro* under darkness for 22DAS, in the presence of HF, IBA, ACC, and IBA+ACC.

Supplementary Fig. 5 Expression pattern of the β-anthranilate synthase gene during AR-formation under HF, IBA, ACC, and IBA+ACC.

Supplementary Table 1. IAA and IBA levels in hypocotyls with ARs excised from wt seedlings grown under HF and with ACC at 14DAS.

Supplementary Fig. 1

HPLC chromatogram (treatment HF) recorded after 8 min (m/z values: IAA = 174.1, IBA = 202.2). The peak labels correspond to the two analyzed phytohormones.

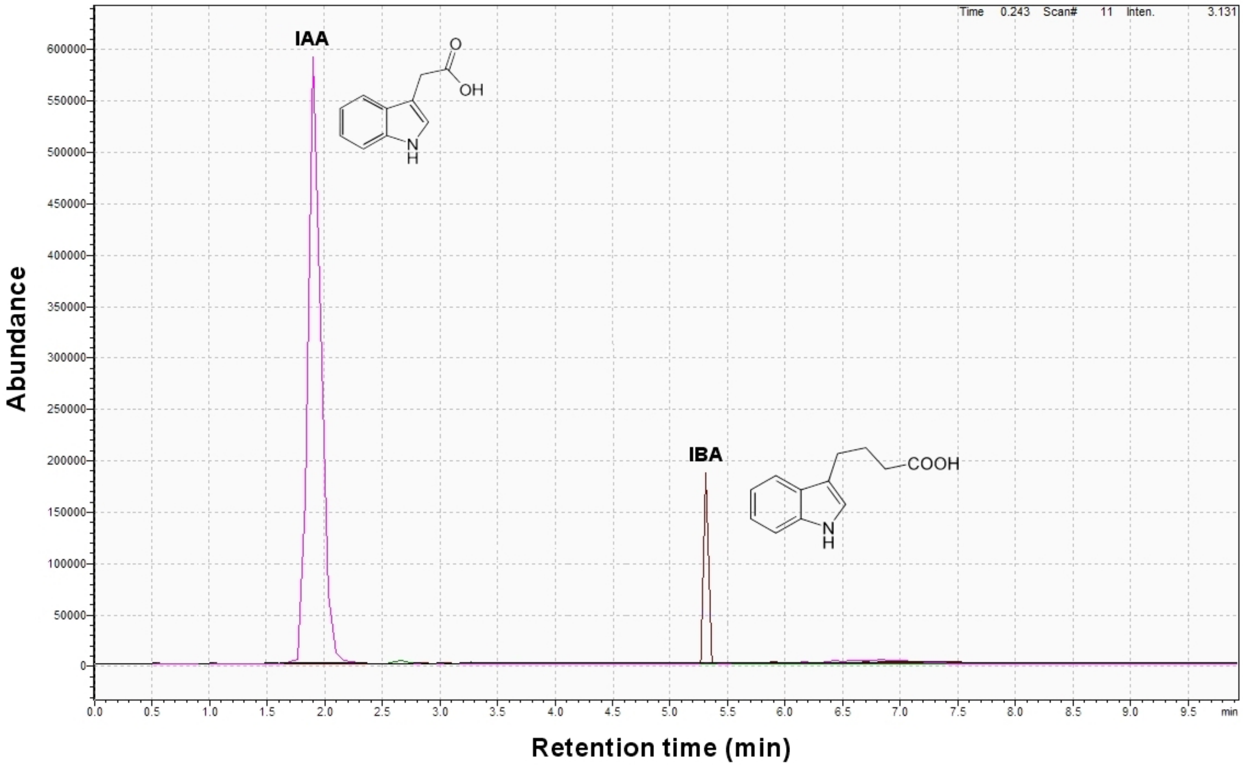

Supplementary Fig. 2 Germination percentage of *Arabidopsis thaliana* seeds, Col-0 ecotype, at 7 DAS in HF condition and in the presence of different ACC concentrations (**A**) or with ACC (0.1µM) and/or IBA (10µM) (**C**), and AR density, i.e., AR-number per cm of hypocotyl, expressed as mean value (±SE), in Col-0 seedlings at 22 DAS, with/without different ACC concentrations (**B)**. *In vitro* growth, under darkness. N=30. c, $P<0.05$ difference with respect to HF. d, $P<0.01$ difference with respect to 0.01 µM ACC. e, $P<0.01$ difference with respect to HF and 0.04µM ACC. Columns with the same letter or no letter are not significantly different. **B** shows a different replicate from the one shown in Fig. 1B. Note that the results are totally similar to those in Fig.1B.

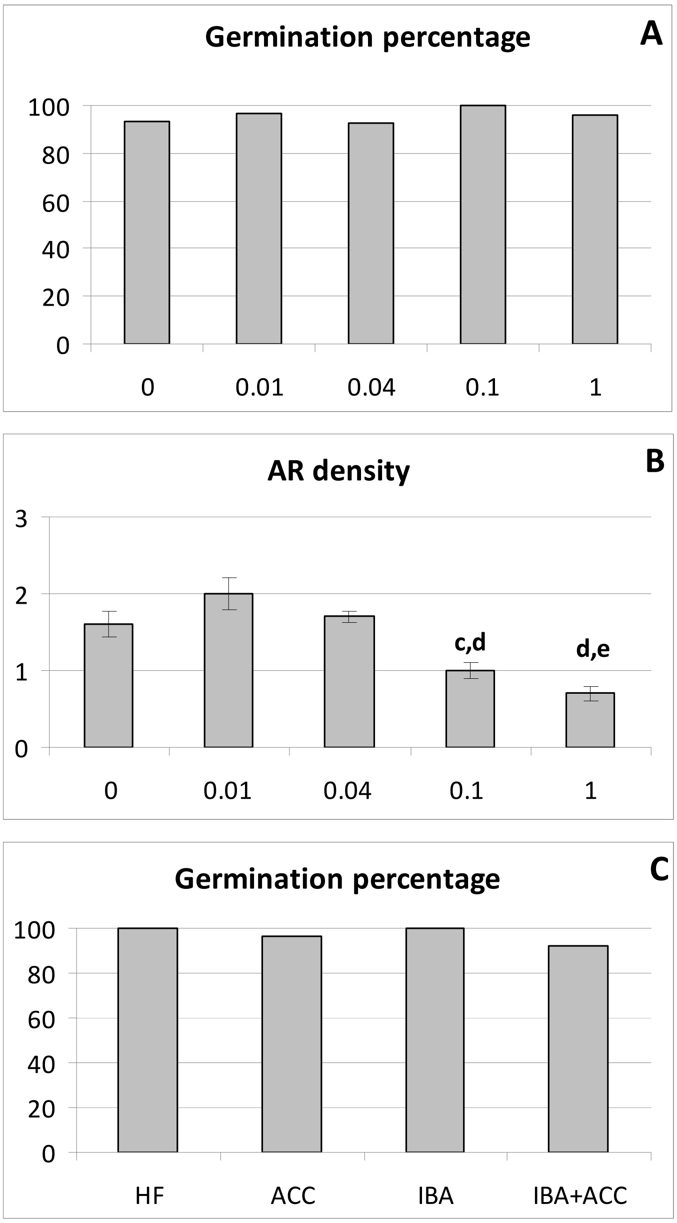

Supplementary Fig. 3 AR-density (±SE) in *Arabidopsis thaliana* seedlings of Col-0 (wt) ecotype and *ein3eil1, wei2wei7* and *ech2ibr10* double mutants at the end of *in vitro* growth (22DAS) under continuous darkness either without hormones (HF) or with ACC (0.1µM) and/or IBA (10µM). N=30. a, *P*<0.01 difference with respect to HF within the same genotype. b, *P*<0.05 difference with respect to HF within the same genotype. c, *P*<0.01 difference with respect to IBA alone within the same genotype. d,e,f, *P*<0.01 difference with respect to the wt within the same treatment. g, *P*<0.05 difference with respect to the wt within the same treatment. h, *P*<0.05 difference with respect to IBA within the same genotype and *P*<0.01 with respect to the wt within the same treatment. N=30. Columns with the same letter, or the asterisk/no letter in different genotypes, are not significantly different. Data of a replicate different from those shown in Figs. 3-5, with totally similar results.

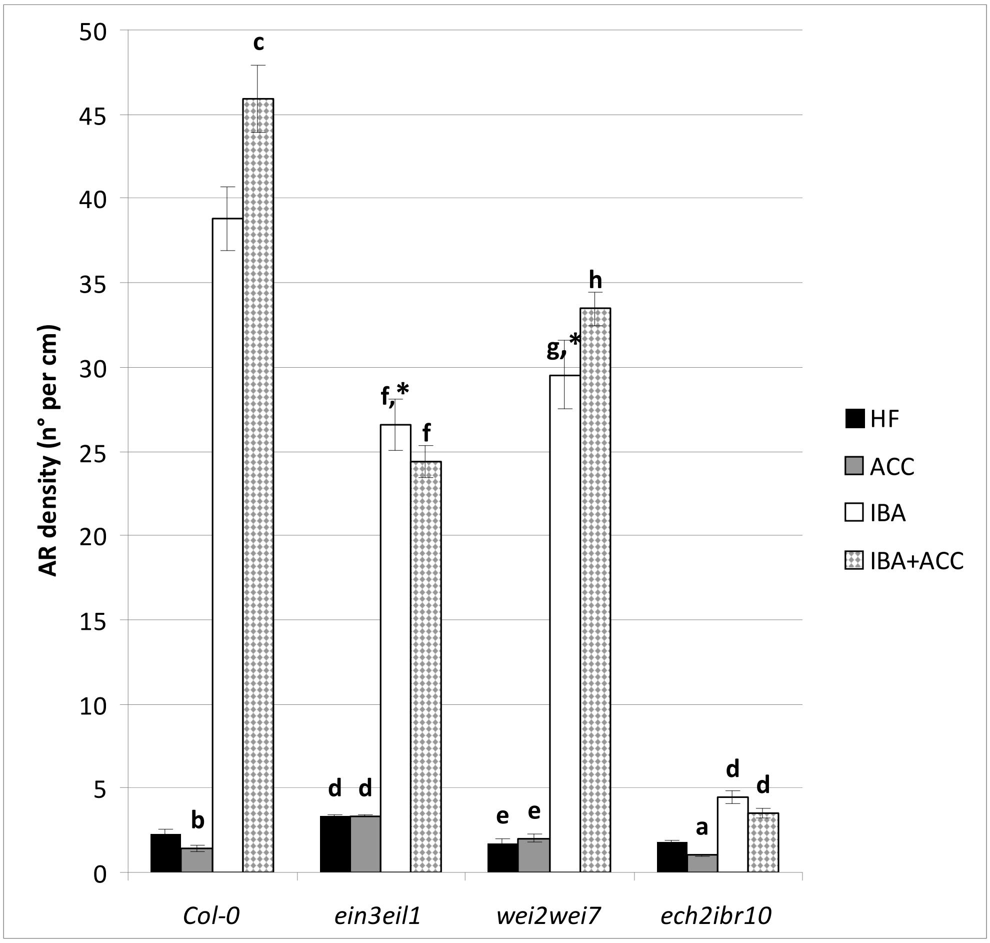

Supplementary Fig. 4 Mean hypocotyl length (±SE) (**A**) and AR-density, i.e., AR-number per cm of hypocotyl expressed as mean value (±SE), (**B**) in *Arabidopsis thaliana* Col seedlings at 22DAS of *in vitro* growth, in continuous darkness, either without hormones (HF), or with ACC (0.1µM) and/or IBA (10µM). N=30. a, $P<0.01$ difference with respect to HF. b, $P<0.01$ difference with respect to IBA alone. Columns with the same letter are not significantly different.

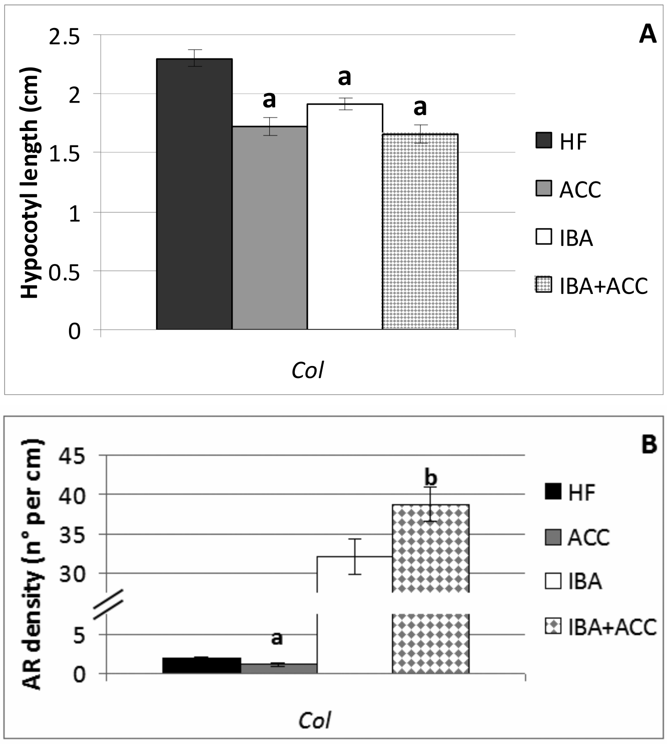

Supplementary Fig. 5 Histochemical GUS analysis of *ASB::GUS* seedlings showing the expression pattern of the β-anthranilate synthase gene during successive phases of AR-formation either under HF (**A-C**), or in the presence of 10μM IBA (**D-E**), or 0.1μM ACC (**F-G**), or both (**H-J**). (Col-0 background, continuous darkness for 22DAS). Bars= 30 μm.

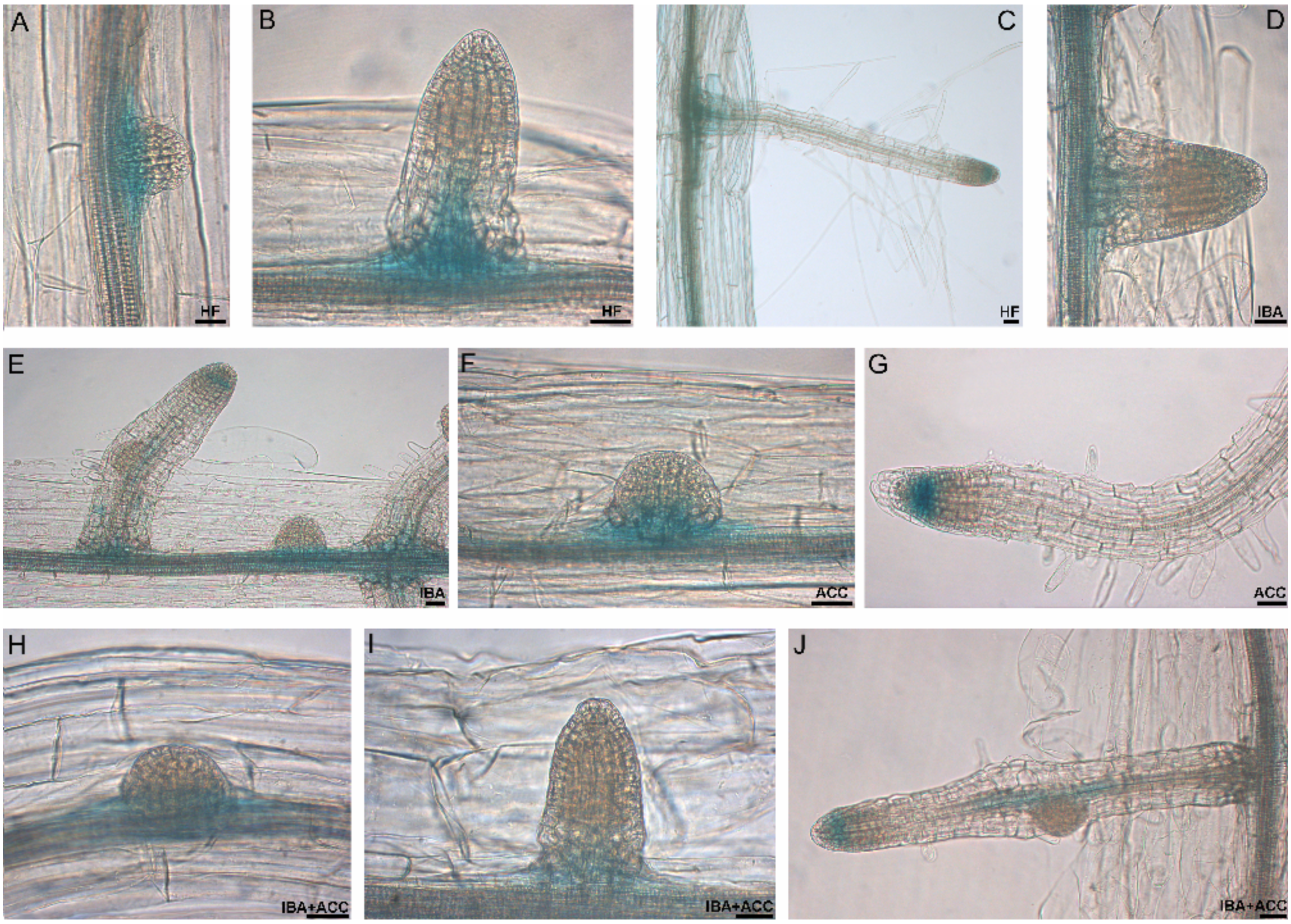

Supplementary Table 1. Mean values (±SE) of IAA and IBA (ng g^{-1} FW) in hypocotyls with ARs excised from Col-0 seedlings after 14 days of growth under darkness either in the absence of exogenous hormones (HF) or in the presence of ACC (0.1 μM). N= 3 replicates per treatment. No significant difference within each column.

Treatments ⇓	**IAA (ng g^{-1} FW)**	**IBA (ng g^{-1} FW)**
**HF**	148.26 ± 2.50	10.85 ± 0.67
**ACC**	161.06 ± 5.00	15.59 ± 0.57
